# Identification of compounds with anti-human cytomegalovirus activity that inhibit production of IE2 proteins

**DOI:** 10.1016/j.antiviral.2016.12.006

**Published:** 2017-02

**Authors:** Rooksarr Beelontally, Gavin S. Wilkie, Betty Lau, Charles J. Goodmaker, Catherine M.K. Ho, Chad M. Swanson, Xianming Deng, Jinhua Wang, Nathanael S. Gray, Andrew J. Davison, Blair L. Strang

**Affiliations:** a Institute of Infection & Immunity, St George's, University of London, London, UK; b MRC-University of Glasgow Centre for Virus Research, Glasgow, UK; c Department of Infectious Diseases, King's College London, London, UK; d Department of Biological Chemistry & Molecular Pharmacology, Harvard Medical School, Boston, MA, USA; e Department of Cancer Biology, Dana-Farber Cancer Institute, Boston, MA, USA

**Keywords:** Human cytomegalovirus, Screen, Kinase, Inhibitor, Compound, IE2

## Abstract

Using a high throughput screening methodology we surveyed a collection of largely uncharacterized validated or suspected kinase inhibitors for anti-human cytomegalovirus (HCMV) activity. From this screen we identified three structurally related 5-aminopyrazine compounds (XMD7-1, -2 and -27) that inhibited HCMV replication in virus yield reduction assays at low micromolar concentrations. Kinase selectivity assays indicated that each compound was a kinase inhibitor capable of inhibiting a range of cellular protein kinases. Western blotting and RNA sequencing demonstrated that treatment of infected cells with XMD7 compounds resulted in a defect in the production of the major HCMV transcriptional transactivator IE2 proteins (IE2-86, IE2-60 and IE2-40) and an overall reduction in transcription from the viral genome. However, production of certain viral proteins was not compromised by treatment with XMD7 compounds.

Thus, these novel anti-HCMV compounds likely inhibited transcription from the viral genome and suppressed production of a subset of viral proteins by inhibiting IE2 protein production.

## Highlights

• High throughput screening identified novel kinase inhibitors that inhibit HCMV protein production.
• 5-aminopyrazine compounds (XMD7-1, -2 and -27) have anti-HCMV activity.
• XMD7 compounds inhibited production of HCMV IE2 proteins.

## 1 Introduction

A number of drugs for the treatment of human cytomegalovirus (HCMV) disease are available for clinical use, including the frontline drug ganciclovir (GCV) (Coen and Schaffer, 2003, Mocarski et al., 2015). However, these drugs have many shortcomings, including the emergence of drug resistant viruses (Coen and Schaffer, 2003).

To develop new anti-HCMV drugs our knowledge of compounds with anti-HCMV activity must be expanded. Identification of compounds that inhibit the function of HCMV proteins essential for HCMV replication would be valuable. However, identification of compounds that inhibit the function of cellular proteins required for HCMV replication should also be explored, as targeting cellular factors may preclude the emergence of drug resistant viruses. Several cellular kinase proteins involved in HCMV replication are required for production of immediate-early (IE) viral proteins IE1 and IE2, an innate and intrinsic immunity antagonist and a transcriptional transactivator, respectively (Mocarski et al., 2015). Production of IE proteins leads to a viral transcriptional cascade (*immediate-early* to *early* to *late* gene transcription) necessary for the production of infectious HCMV (Mocarski et al., 2015). Therefore, inhibition of cellular protein kinases could lead to reduced IE protein production and HCMV replication. However, our understanding of how cellular protein kinases are involved in HCMV replication is incomplete and it is likely that several kinase proteins required for IE protein production have to yet be identified.

We have previously developed a high throughput siRNA screening methodology to identify siRNAs that positively or negatively influence HCMV protein production (Polachek et al., 2016). Here, we adapted this methodology to identify compounds that inhibit HCMV protein production. We then sought to expand our understanding of compounds that can be developed to anti-HCMV drugs by applying our screen to the Gray Kinase inhibitor library; a collection of compounds, many of which have not been previously characterized, comprising validated and suspected ATP-site kinase inhibitors that target active and inactive kinase conformations of cellular kinase proteins.

## 2 Materials and methods

### 2.1 Compounds

The Gray Kinase Inhibitor library was supplied to the Institute of Chemistry and Chemical Biology-Longwood at Harvard Medical School by Nathanael S Gray. Any available information on compounds within the Gray Kinase Inhibitor library can be found at the Harvard Medical School (HMS) LINCS online resource (http://lincs.hms.harvard.edu/), which is part of the National Institutes of Health Library of Integrated Network-based Cellular Signatures (LINCS) Program or is available by request from Nathanael S Gray. All drugs were resuspended in dimethyl sulfoxide (DMSO).

### 2.2 Cells and viruses

Human foreskin fibroblast (HFF) cells (clone Hs29) were obtained from American Type Culture Collection no. CRL-1684 (ATCC, Manassas, VA) and maintained in Dulbeccos modified Eagles medium (DMEM) (Gibco) containing 5% fetal bovine serum (FBS) (Gibco), plus penicillin and streptomycin. High passage HCMV strain AD169 was a gift from Don Coen (Harvard Medical School). Low passage strain Merlin RCMV1111 (derived from BACmid pAL1111, which does not express RL13 and UL128) (Stanton et al., 2010) was a gift from Richard Stanton (Cardiff University).

### 2.3 High throughput screening of compounds

See Supplementary Material.

### 2.4 Synthesis of XMD7 compounds

See Supplementary Material.

### 2.5 Viral yield reduction assays

5 × 10^4^ HFF cells per well were plated in 24-well plates. Cells were incubated overnight and then infected with HCMV at a multiplicity of infection (MOI) of 1. Virus was adsorbed to cells for 1 h at 37 °C and then cells were incubated with 0.5 ml of media containing DMSO or compounds at a range of concentrations in duplicate. Plates were incubated for 96 h at 37 °C. Viral titre (plaque forming units (p.f.u.) per ml) was determined by titration of viral supernatants on HFF monolayers. The mean value of duplicate plaque counts was calculated. The final concentration of DMSO in all samples was maintained at <1% (v/v).

### 2.6 MTT assays

1 × 10^4^ HFF cells per well were plated in 96-well plates. Cells were incubated overnight and then treated for the time indicated in the text with compounds at range of concentrations in duplicate. Relative cell number was then determined with an MTT assay according to the manufacturer's instructions (GE Healthcare). The mean value of duplicate readings was calculated. The final concentration of DMSO in all samples was maintained at <1% (v/v). As a positive control, in all experiments a 2-fold dilution series of HFF cells starting at 1 × 10^4^ cells per well was included. In each experiment we found a linear relationship between the number of cells per well and output from the MTT assay (data not shown).

### 2.7 Kinase selectivity analysis

Each compound was submitted to DiscoverX for Ambit KIMOMEscan analysis on a panel of 353 kinase proteins in the presence of 10 μM of each compound. Information concerning each kinase protein assayed can be found at www.discoverx.com.

### 2.8 Western blotting

At time points indicated in the text cells were washed once with PBS and resuspended in 100 μl Laemmli buffer containing 5% β-mercaptoethanol. Proteins were separated on 8% or 10% polyacrylamide gels. Membranes were probed with antibodies recognizing IE1/2, pp28, pp65, UL44, UL84, (Virusys, 1:1000 dilution), IE2 proteins (clone 5A8.2, Millipore, 1:1000 dilution) and β-actin (SIGMA, 1:5000 dilution). All primary antibodies were detected using anti-mouse- or anti-rabbit-horseradish peroxidase (HRP) conjugated antibodies (Millipore and Cell Signaling Technology, respectively). Chemiluminescence solution (GE Healthcare) was used in each case to detect secondary antibodies using film. Relative band intensity (band intensity relative to β-actin signal in the same lane) was analyzed using ImageJ software, obtained from the National Institutes of Health (USA).

### 2.9 Preparation of RNA for sequencing

5 × 10^6^ HFF cells were infected with HCMV strain AD169 at an MOI of 1 in the presence of DMSO or 1 μM XMD7-1. At 24, 48 and 72 h.p.i., cells were trypsinized, pelleted and stored at −80 °C. Total infected cell RNA was isolated by using an RNeasy Midi Kit (Qiagen). To generate sequencing libraries RNA was prepared using either a Globin-Zero Gold rRNA Removal Kit to remove rRNA, or a module of an Illumina TruSeq Stranded mRNA Library Prep Kit to select poly(A) RNA, followed by the remaining modules of an Illumina TruSeq Stranded mRNA Library Prep Kit.

### 2.10 RNA sequencing and analysis

The sequencing libraries were analyzed on an Illumina MiSeq, collecting single-end data sets of 75 nucleotides. The sequence of HCMV strain AD169 in GenBank accession BK000394 was used to align read data and specify sequences representing exon-exon junctions. Whole genome transcriptome profiles were visualized by using Artemis (Carver et al., 2012), and reads representing exon-exon junctions were enumerated by using the Unix grep program.

## 3 Results

### 3.1 High throughput screening of a compound library

**Fig. 1:**
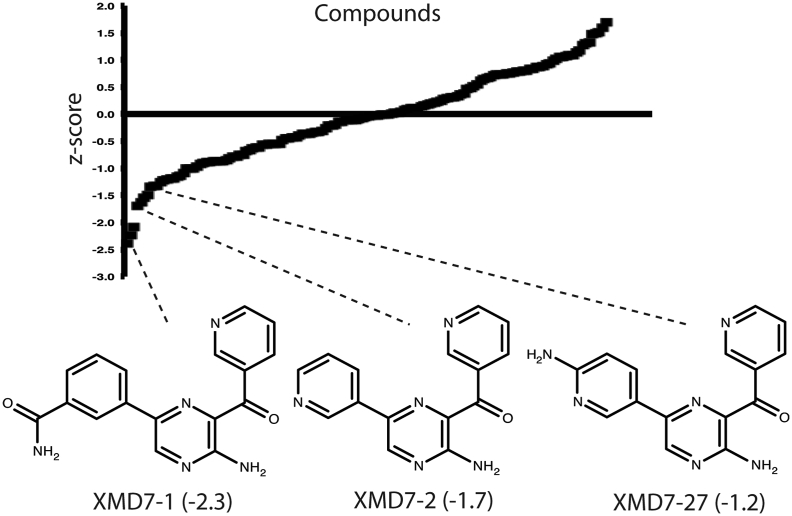
**Compounds assigned z-scores.** Plot of z-scores where each data point represents a single compound. A list of each compound with its assigned z-scores is shown in Supplementary Table S3. The structures and z-scores (stated in parentheses) of XMD7-1, -2 and -27 are identified.

We screened the Gray Kinase Inhibitor library of 187 compounds (Table S1) for anti-HCMV activity using a methodology based on our previous siRNA screening experiments (Polachek et al., 2016). Briefly, HFF cells were treated with each compound from the collection and infected with HCMV strain AD169. Cells were then stained with antibodies to detect the HCMV antigen pp28. As pp28 is expressed late in infection, detection of pp28 allowed our screen to identify compounds affecting all stages of HCMV replication from entry of virus into the cell to late gene expression. The total number of cells and the number of infected cells were enumerated using automated microscopy. In wells where the mean number of cells was 2-fold below the mean number of cells of the plate, the compound in that well was judged to be cytotoxic (41 compounds, Table S2). Data from compounds not excluded for cytotoxicity were converted to a z-score (the number of standard deviations from the mean of the data (Birmingham et al., 2009, Zhang et al., 1999)) to show an increase or decrease (positive or negative z-score, respectively) in the number of pp28 positive cells (146 compounds, Fig. 1 and Table S3).

The “hit” in our screen with the greatest negative z-score was a previously uncharacterized 5-aminopyrazine compound, XMD7-1 (Fig. 1). We also found that two other previously uncharacterized 5-aminopyrazine compounds structurally related to XMD7-1, XMD7-2 and XMD7-27, were assigned negative z-scores (Fig. 1).

### 3.2 Inhibition of HCMV replication by XMD7 compounds

To confirm the anti-HCMV activity of XMD7 compounds, we used viral yield reduction assays to assess replication of high passage HCMV strain AD169 and low passage HCMV strain (Merlin (RCMV1111)), whose genomic content is similar to that of a clinical virus (Wilkinson et al., 2015), in the presence of XMD7 compounds. The 50% effective dose (ED50) of each compound against AD169 and Merlin was 0.3–0.4 μM and 2.1–2.5 μM, respectively. Therefore, XMD7 compounds were inhibitors of HCMV replication and had anti-HCMV activity against different strains of HCMV.

To ensure that the anti-HCMV activity of XMD7 compounds was not due to cellular cytotoxicity or inhibition of cell division, we used an MTT assay to assess relative cell number in uninfected cells in the presence of these compounds compared to treatment of cells with DMSO. We found that the 50% cellular cytotoxicity (CC50) of each XMD7 compound was greater than 10 μM (Table 1). Therefore, the CC50 of each compound was at least 10-fold greater than the ED50 of compounds. Thus, the observed anti-HCMV activity of XMD7 compounds was unlikely to have been due to their cytotoxicity or inhibition of cell division. Consistent with this conclusion, we observed no obvious cell death or changes to cell morphology at any time after treatment of uninfected cells with XMD7 compounds (data not shown).

**Table 1:** Anti-viral activity and cytotoxicity of XMD7 compounds.

Assay	Viral Strain	Compound	ED50^a^	CC50^a^
Viral Yield Reduction^b^	AD169	XMD7-1	0.3	–
	AD169	XMD7-2	0.3	–
	AD169	XMD7-27	0.4	–
Viral Yield Reduction^b^	Merlin (RCMV1111)	XMD7-1	2.5	–
	Merlin (RCMV1111)	XMD7-2	2.1	
	Merlin (RCMV1111)	XMD7-27	2.4	
MTT Cytotoxicity^c^	–	XMD7-1	–	>10
	–	XMD7-2	–	>10
	–	XMD7-27	–	>10

a μM.

b Viral titre was assessed at 96 h post infection in the presence of each compound.

c MTT assays were carried out after 96 h exposure of cells to each compound.

### 3.3 Kinase selectivity analysis of XMD7 compounds

**Fig. 2:**
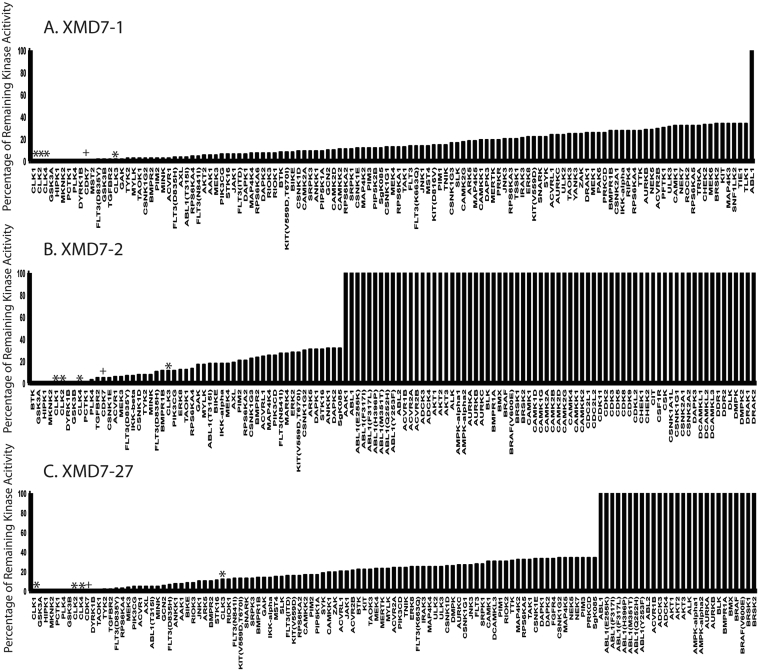
**Kinase selectivity of XMD7 compounds**. (A)–(C) Each compound (XMD7-1, -2 and -27, respectively) was subjected to Ambit KINOMEScan analysis. Each figure shows the 123 kinase proteins most potently inhibited by each compound. Full lists of kinase inhibition data for XMD7-1, -2 and -27 are shown in Tables S4–S6, respectively. Asterisks and crosses denote the ability of each compound to inhibit CLK proteins or CDK7, respectively.

We then sought to understand whether XMD7 compounds were kinase inhibitors and to determine which kinase proteins they could inhibit. We subjected each compound to Ambit KINOMEScan analysis, wherein the ability of each compound to inhibit a panel of 353 cellular kinase proteins was assayed. We found that each XMD7 compound inhibited a similar range of kinase proteins, albeit with different potencies (Fig. 2A–C and Tables S4–S6). Therefore, inhibition of HCMV replication in the presence of XMD7 compounds was likely related to their ability to inhibit the function of the cellular kinase proteins shown in Fig. 2A–C.

Furthermore, we used western blotting to investigate autophosphorylation of the HCMV encoded kinase UL97 in the presence of XMD7-1. UL97 autophosphorylation was not affected by XMD7-1 (data not shown). Therefore, the anti-HCMV activity of XMD7 compounds was unlikely to have been due to inhibition of UL97.

### 3.4 Examination of events early in HCMV replication

We then sought to understand how XMD7 compounds inhibit HCMV replication. To investigate if XMD7-1 could affect virus entry into the cell or translocation of the HCMV genome to the nucleus, we treated AD169 infected HFF cells with 1 μM XMD7-1 at 24 h.p.i. We found an approximately 2-fold decrease in HCMV replication at 120 h.p.i., compared to infected cells treated with DMSO at 24 h.p.i. (data not shown). Therefore, XMD7-1 could inhibit HCMV replication after HCMV entry and the inhibitory effects of XMD7-1 were likely related to inhibition of events after entry of the virus into the cell.

**Fig. 3:**
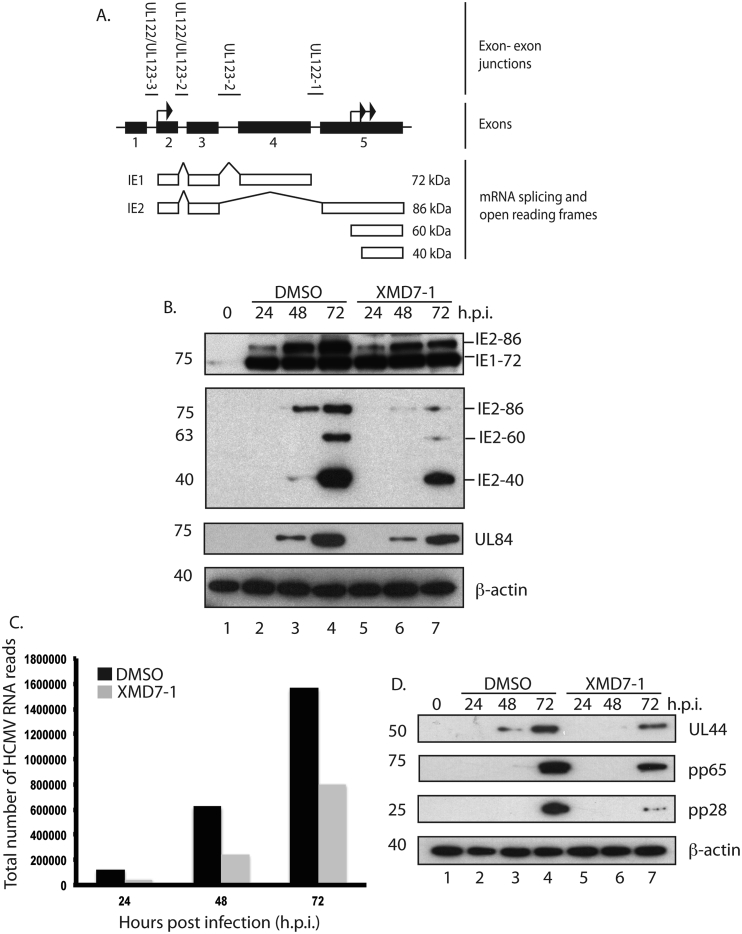
**Western blotting and RNA sequencing from HCMV infected cells treated with XMD7-1**. (A) Organization of HCMV sequences encoding IE1/2 proteins and IE1/2 proteins produced during HCMV replication. The 5 exons of the HCMV UL122-123 locus that encode IE1 and IE2 proteins are shown. Black arrows in exons 2 and 5 represent start codons. Above the exons are the exon-exon junctions. Below the exons the alternative spicing of RNAs that encode IE1 and IE2 proteins is shown, as are IE2 proteins IE2-60 and IE2-40 produced from internal start sites in exon 5. (B and D) HFF cells were infected with AD169 at an MOI of 1, then treated with either 1 μM XMD7-1 or the equivalent volume of DMSO at the time of infection. Cell lysates were prepared for western blotting at the time points (hours post infection (h.p.i.)) indicated above the figure. Uninfected cells harvested at the time of infection are shown as 0 h.p.i. Proteins recognized by the antibodies used are indicated to the right of each figure. The positions of molecular mass markers (kDa) are indicated to the left of each figure. (C) Number of HCMV RNA sequence reads from total RNA (excluding rRNA) from HFF cells infected with AD169 at an MOI of 1, then treated with either 1 μM XMD7-1 or the equivalent volume of DMSO at the time of infection.

Next, we used western blotting to assay HCMV IE protein production in the presence of XMD7-1. IE1-72 and IE2-86 are produced by alternative splicing of mRNA transcripts produced from the UL122-123 locus (Fig. 3A). Other IE2 proteins, IE2-60 and IE2-40, are produced from internal start codons within exon 5 of the UL122-123 locus (Fig. 3A). When comparing protein production in the presence of XMD7-1 (Fig. 3B, lanes 5–7) to treatment with DMSO (Fig. 3B, lanes 2–4), we found that XMD7-1 had no effect on the production of IE1-72, but resulted in a 3-fold and 6-fold decrease in IE2-86 at 72 h.p.i. (Fig. 3B, top and second to top panels, respectively) and a 9- and 2-fold reduction in production of IE2-60 and IE2-40 72 h.p.i., respectively. Similar results were observed when infected cells were treated with XMD7-2 or XMD7-27 (data not shown). Therefore, the anti-HCMV activity of XMD7 compounds was associated with suppression of IE2 protein production.

### 3.5 Analysis of HCMV RNA production and splicing

We then considered why IE2, but not IE1, protein production was inhibited in the presence of XMD7-1. We found that each XMD7 compound was a potent inhibitor of CLK proteins -1, -2, -3 and -4 (indicated with asterisks in Fig. 2A–C) and treatment of HCMV infected cells with XMD7-1 resulted in an increase in CLK-1 protein (data not shown), which is consistent with XMD7-1 acting as a CLK kinase inhibitor (Ninomiya et al., 2011). RNA splicing is modulated by the SRSF family of proteins and SRSF protein function can be influenced via phosphorylation by CLK proteins. Therefore, we hypothesized that treatment of HCMV infected cells with XMD7-1 resulted in suppression of SRSF phosphorylation and de-regulation of the alternative mRNA splicing producing transcripts encoding either IE1 or IE2.

We addressed SRSF phosphorylation using western blotting. A decrease in phosphorylation of SRSF protein SRSF4, but not any other SRSF protein, was observed (data not shown). However, treatment of HCMV infected cells with siRNA targeting mRNA encoding SRSF4 had no obvious effect on production of infectious HCMV (data not shown). Therefore, the anti-HCMV effect of XMD7-1 was unlikely to involve inhibition of SRSF4 phosphorylation.

To assess viral RNA production and splicing, total RNA was isolated from HCMV infected cells treated with XMD7-1 and, after removal of rRNA, RNA was sequenced and the sequence reads were aligned to the genomic sequence of AD169. We found approximately 2-fold fewer total number of HCMV sequences reads in cells treated with XMD7-1 compared to cells treated with DMSO (Fig. 3C) across the entire HCMV genome (data not shown). We then analyzed the RNA sequence reads for the presence of a selection of exon-exon junctions within HCMV RNA transcripts (Gatherer et al., 2011). Treatment with XMD7-1 resulted in a general decrease in the number of reads representing junctions, including those encoding IE1 or IE2 (Fig. 3A) at 24, 48 and 72 h.p.i. (Table S7), which likely parallels the reduction in the total number of HCMV sequence reads (Fig. 3C). To ensure that this analysis was not biased by the method of RNA sample preparation, we performed a similar analysis of the same total RNA samples from which poly(A) RNA had been selected. Similar results were obtained in all respects (data not shown). Therefore, the reduction in IE2 proteins in cells treated with XMD7-1 was associated with reduction in RNA transcription across the entire HCMV genome and not with reduction of specific exon-exon junctions required for production of IE2 mRNAs.

### 3.6 Examination of CDK inhibition by XMD7 compounds

Production of IE2 proteins, but not IE1, is compromised when HCMV infected cells are treated with the broad-spectrum cyclin-dependent kinase (CDK) inhibitor roscovitine (Sanchez and Spector, 2006), suggesting that CDK inhibition and cell cycle arrest are related to inhibition of IE2 production. We found that XMD7 compounds inhibited CDK7 (Fig. 2A–C, indicated with crosses), but not other CDK proteins (Tables S4–S6), and that treatment of uninfected cells with XMD7 compounds had no effect of cell proliferation as determined using MTT assays (data not shown). Therefore, CDK inhibition upon treatment of infected cells with XMD7 compounds was unlikely to be related to the anti-HCMV activity of XMD7 compounds.

### 3.7 Investigation of HCMV protein production

We then investigated how suppression of HCMV transcription and IE2 protein production in the presence of XMD7-1 affected production of certain early (UL44), delayed early (UL84 and pp65) and late (pp28) HCMV proteins. IE2-86 is required for initiation of the viral transcriptional cascade and, therefore, is required for expression of all viral proteins (Heider et al., 2002). Moreover, IE2-86 and IE2-40 are required for the post-translational stabilization of UL84 (Sanders et al., 2008, Sanders and Spector, 2010) and deletion of IE2-60 and IE-40 results in suppression of pp65 production (White et al., 2007). IE2-60 and IE2-40 are not required for efficient expression of either UL44 or pp28 (White et al., 2007). Using western blotting we found less than 2-fold difference in accumulation of UL84 (Fig. 3B), but a 2-fold decrease in both UL44 and pp65 expression and a 10-fold decrease in pp28 expression (Fig. 3D), in HCMV infected cells treated with XMD7-1 compared to HCMV infected cells treated with DMSO. We found similar phenotypes when lysates of infected cells treated with either XMD7-2 or XMD7-27 were assayed (data not shown). Therefore, treatment of infected cells with XMD7 compounds had no obvious effect on IE1 or UL84 production, but resulted in suppression of all other HCMV proteins assayed with the reduction in pp28 production being greater than that of any other protein. Thus, treatment with XMD7 compounds inhibited transcription across the entire HCMV genome, but did not compromise production of all HCMV proteins.

## 4 Discussion

Our screen identified a number of compounds that positively or negatively influenced HCMV protein production and also identified a number of compounds that are cytotoxic to HCMV infected cells. As the compounds that were screened are largely uncharacterized, further mining of our data will identify the relationship between compound structures, compound targets, anti-HCMV activity and cytotoxicity.

The XMD7 compounds are novel anti-HCMV compounds that can efficiently inhibit HCMV replication. To our knowledge this is the first report identifying 5-aminopyrazine compounds with anti-HCMV activity. No other compounds structurally related to XMD7 were assayed in our screen. Therefore, further 5-aminopyrazine compounds must be assayed to discover the structure-activity relationship that dictates the anti-HCMV activity of XMD7 compounds. This will also allow design of compounds with improved anti-HCMV activity and will aid identification of kinase targets required for anti-HCMV activity.

We found that the XMD7 compounds display polypharmacology. Each of the kinase proteins potently inhibited by XMD7 compounds will have to be assayed alone and in combination to discover the anti-HCMV target of the XMD7 compounds. Many of these kinase proteins have not previously been reported to be involved in HCMV replication. Therefore, assaying the requirement for many of these kinase proteins during HCMV replication may identify cellular kinase proteins hitherto not recognized to be involved in HCMV replication.

We propose that the mechanism of action of XMD7 compounds involves inhibiting production of IE2 proteins, which leads to a defect in transcription from the HCMV genome and a defect in production of certain HCMV proteins (UL44, pp65 and pp28, but not IE1 or UL84). This is somewhat consistent with an essential role for IE2-86 in viral transcriptional transactivation (Heider et al., 2002). However, it is unclear why production of all HCMV proteins is not compromised. IE2-60 and IE2-40 have direct roles in production of UL84 (Sanders et al., 2008, Sanders and Spector, 2010). It is possible that there was sufficient production of IE2 proteins to maintain efficient production of UL84 in the presence of XMD7 compounds. However, it is unclear why levels of IE1 were not affected. Also, a notable observation was that suppression of pp28 production was greater than that observed for UL44 and pp65. Deletion of HCMV protein UL26 from the HCMV genome results in hypophosphorylation of pp28 during HCMV replication, which results in instability of pp28 (Munger et al., 2006). Thus, it is possible that treatment of HCMV infected cells with XMD7 compounds also leads to inhibition of UL26 function, or inhibition of the as yet unidentified kinase responsible for pp28 phosphorylation, which results in suppression of pp28 production.

6-aminoquinolone compounds (Loregian et al., 2010, Massari et al., 2013, Mercorelli et al., 2014) and repurposed bioactive small molecules (Mercorelli et al., 2016) that inhibit IE2 transcriptional transactivation and have anti-HCMV activity have been reported. These compounds are structurally dissimilar to XMD7 compounds and, in contrast to XMD7 compounds, do not inhibit IE2 production in HCMV infected cells (Loregian et al., 2010, Mercorelli et al., 2016). Therefore, these compounds have a different mechanism of action compared to XMD7 compounds. These previously identified compounds and our present work underscore that targeting IE2 production or function is a valid route to inhibiting HCMV replication. Further development of these compounds will illuminate the molecular basis of their action, including the direct or indirect effects these compounds have on viral replication and cell function.

## Funding

We would like to express our thanks to Don Coen for his encouragement during this study and his support of B.L.S. through grants awarded to D.C. from the 10.13039/100000002National Institutes of Health (R01 AI019838 and R01 AI026077). This work was also supported by New Investigator funds from St George's, University of London, a St George's Impact & Innovation Award and a PARK/WestFocus Award (all to B.L.S.) and by Medical Research Council grant MC_UU_12014/3 (to A.J.D.). The funders had no role in experimental design, data collection, data interpretation or the decision to submit the work for publication.

## Appendix A Supplementary data

**Supplementary Material.** High throughput screening and synthesis of XMD7 compounds.

**Supplementary Table S1.** List of compounds within the Gray Kinase Inhibitor library.

**Supplementary Table S2.** List of compounds judged to be cytotoxic in the screening process.

**Supplementary Table S3.** List of compounds assigned z-scores from analysis of screening data.

**Supplementary Table S4.** AMBIT KinomeScan profile of XMD7-1.

**Supplementary Table S5.** AMBIT KinomeScan profile of XMD7-2.

**Supplementary Table S6.** AMBIT KinomeScan profile of XMD7-27.

**Supplementary Table S7.** Analysis of HCMV splice sites found in RNA sequencing (rRNA purified).
