# Supplementary material for: Identification of compounds with anti-human cytomegalovirus activity that inhibit production of IE2 proteins
Source: Antiviral Res. 2017 Feb;138:61–7. doi: 10.1016/j.antiviral.2016.12.006 (PMC5244968; doi:10.1016/j.antiviral.2016.12.006)
Supplement: Supplementary file 1 [file mmc1.docx]

**SUPPLEMENTARY MATERIAL**

Identification of Compounds with Anti-Human Cytomegalovirus Activity that Inhibit Production of IE2 Proteins

Rooksarr Beelontally^1^, Gavin S Wilkie^2^, Betty Lau^2^, Charles J Goodmaker^1^, Catherine M K Ho^1^, Chad M Swanson^3^, Xianming Deng^4,5^, Jinhua Wang^4,5^, Nathanael S Gray^4,5^, Andrew J Davison^2^ & Blair L Strang^1,4^

Institute of Infection & Immunity, St George’s, University of London, London, UK^1^; **MRC-University of Glasgow Centre for Virus Research**, Glasgow, UK^2^; Department of Infectious Diseases, King's College London, London, UK^3^; Department of Biological Chemistry & Molecular Pharmacology, Harvard Medical School, Boston, MA, USA^4^**;** Department of Cancer Biology, Dana-Farber Cancer Institute, Boston, MA, USA^5^.

**Compound treatment and infection of cells for high throughput screening.** The Gray Kinase Inhibitor library (stock concentration of 3.3 mM of each compound in DMSO) was screened in duplicate. Twenty four hours before infection 2 × 10^3^ HFF cells were seeded in each well of each Corning 384 plate. Unless stated otherwise, liquid was added to wells using a WellMate apparatus. At the time of infection, medium was removed with a suction manifold and 30 μl of complete medium was added to each well. Compounds were added to the plate containing HFF cells using a 100 nl pin transfer on a liquid handling robot. Negative and positive controls (water+0.3% DMSO or heparan sulfate (5 μg/ml) + 0.3% DMSO, respectively) were added to plates by hand (12 wells of each). Cells were then infected with HCMV strain AD169 (multiplicity of infection (MOI) of 1 plaque-forming unit (p.f.u.) per cell) in a total volume of 5 μl. Thus, the final concentration of compound in each well was 9.4 μM. Infected cells were incubated for 72 hours at 37^o^C and then prepared for analysis.

**Preparation of screening plates for high throughput microscopy analysis.** Cell culture medium was removed from infected cells and replaced with 20 μl Hoecsht 33342 (SIGMA) diluted in PBS to a final concentration of 10 μg/ml. After incubation for 1 hour at 37^o^C, 20 μl of Deep Red Cell Mask (Invitrogen) (diluted in PBS to a concentration of 5 μg/ml) was added to each well. Cells were incubated for a further 5 min at 37^o^C. Cells were then fixed by removing PBS containing Hoescht and Cell Mask and adding 50 μl of 3.5%Formaldyhyde (SIGMA) in PBS to each well. After incubating at room temperature for 10 min, fixative was removed and 50 μl of PBS containing 0.5% TritonX-100 was added per well to permeablize cells. After 10 min incubation at room temperature, PBS containing detergent was removed, and cells were washed once with PBS. PBS was removed and replaced with 20 μl MAb P207 recognizing pp28 (Virusys) (dilution 1:1000) and anti-mouse secondary antibody conjugated to flurophore Alexa488 (Molecular Probes) (dilution 1:1000). Plates were incubated at 37^o^C for 1 hour. After incubation, PBS containing antibodies was removed and replaced with 50 μl of PBS. Plates were then analyzed using automated microscopy for the presence of pp28 protein.

**Microscopy analysis of screening plates.** Infected cells stained with antibody to detect pp28 were imaged on an Image Express Micro (IXM) microscope (Molecular Devices) at 10x magnification to detect 3 wavelengths; 488 nm to detect antibody recognizing pp28, 568nm to detect Deep Red CellMask and 350 nm to detect Hoescht 33342 stain bound to nuclear DNA. Three images were captured from each wavelength in each well of the 384-well plate. The number of cells positive at all 3 wavelengths and percentage of pp28 positive cells in each well were determined using the Metamorph Multiwavelength Cell Scoring software (Molecular Devices). Typically, approximately 60% of cells were infected in wells treated with negative control, DMSO (data not shown).

**Analysis of screening results.** To assess the quality of data that could be returned from the screening protocol we calculated the Z’-factor ([Birmingham et al., 2009](#_ENREF_1); [Zhang et al., 1999](#_ENREF_2)) derived from the positive (heparan sulphate treated infected cells) and negative (DMSO treated infected cells) control wells. The screening controls returned Z’-factors of greater than or equal to 0.5, indicating a robust separation of difference in the data derived from positive and negative controls (data not shown). Thus, the screening protocol could be reliably used to screen the compound collection.

After screening of the compound collection, data were discarded from any well in which the number of cells stained with Hoescht 33342 fell below 2-fold of the mean of the number of cells in each well of the plate. The data from the remaining wells from each plate was converted to a z-score (the number of standard deviations from the mean of the data ([Birmingham et al., 2009](#_ENREF_1); [Zhang et al., 1999](#_ENREF_2))) and the average z-score from data in duplicate plates was determined. Images chosen at random were visually inspected throughout image capture and analysis to ensure raw data were consistent with z-scores.

**Chemistry and synthetic procedure for XMD7 compounds.** XMD7-1 and XMD7-2 were synthesized based on synthesis of XMD7-27 in patent WO2009115517. Unless otherwise noted, reagents and solvents were obtained from commercial suppliers and were used without further purification. ^1^H NMR spectra were recorded on 600 MHz (Varian AS600), and chemical shifts are reported in parts per million (ppm, δ) downfield from tetramethylsilane (TMS). Coupling constants (*J*) are reported in Hz. Spin multiplicities are described as s (singlet), d (doublet). Mass spectra were obtained on a Waters Micromass ZQ instrument. Preparative HPLC was performed on a Waters Symmetry C18 column (19 x 50 mm, 5µM) using a gradient of 5-95% acetonitrile in water containing 0.05% trifluoacetic acid (TFA) over 8 min (10 min run time) at a flow rate of 30 mL/min. Purities of assayed compounds were in all cases greater than 95%, as determined by reverse-phase HPLC analysis.

Reagents and conditions: a) 2,2,6,6-Tetramethyl piperidine, n-Butyllithium, THF, -78^o^C - r.t.; b) Manganese dioxide, Toluene, 110 ^o^C; c) ammonia (2.0 M in isopropanol), 100 ^o^C; d) bromine, acetic acid, sodium carbonate, r.t..

1) Intermediates **1** - **4** were synthesized based on PCT WO2009115517.

# 2) General synthetic procedure of XMD7-1 and XMD7-2:

# (3-amino-6-bromo-2-pyrazinyl)-3-pyridinyl-Methanone (1 eq.), β-[3-(aminocarbonyl)phenyl]-Boronic acid (1.3 eq) or β-3-pyridinyl-Boronic acid (1.3 eq), [1,1′-Bis(diphenylphosphino)ferrocene]dichloropalladium (0.1 eq.), and 1M aqueous Na_2_CO_3_ (5 eq.) were added into a mixture of toluene and ethanol (6:1). The reaction was stirred at 100 °C for 2h after degas. The reaction mixture was diluted with ethyl acetate and filtered through a Celite pad, washed with water and saturated brine solution, and then dried with anhydrous sodium sulfate. Purification by HPLC provided final product:

3-[5-amino-6-(3-pyridinylcarbonyl)-2-pyrazinyl]-Benzamide (XMD7-1)

^1^H NMR (600 MHz, CD_3_OD) δ 9.38 (s, 1H), 8.95 (s, 1H), 8.87 (s, 1H), 8.85 (s, 1H), 8.39 (s, 1H), 8.06 (s, 1H), 7.95 (s, 1H), 7.86 (s, 1H), 7.54 (s, 1H). MS (ESI) *m/z* 320.16 (M+H)^+^.

[3-amino-6-(3-pyridinyl)-2-pyrazinyl]-3-pyridinyl-Methanone (XMD7-2)

^1^H NMR (600 MHz, CD_3_OD) δ 9.38 (s, 2H), 9.15 (s, 1H), 8.94 (s, 1H), 8.83 (s, 1H), 8.77 (d, J=8.4 Hz, 1H), 8.67 (d, *J* = 7.2 Hz, 1H), 7.91 (s, 1H), 7.84 (s, 1H). MS (ESI) *m/z* 278.10 (M+H)^+^.

Birmingham, A., et al., 2009. Statistical methods for analysis of high-throughput RNA interference screens. Nature methods 6, 569-575.

Zhang, J.H., et al., 1999. A Simple Statistical Parameter for Use in Evaluation and Validation of High Throughput Screening Assays. Journal of biomolecular screening 4, 67-73.
